# Exploring expectations and perceptions of different manual therapy techniques in chronic low back pain: a qualitative study

**DOI:** 10.1186/s12891-021-04251-3

**Published:** 2021-05-14

**Authors:** A. Plank, A. Rushton, Y. Ping, R. Mei, D. Falla, N. R. Heneghan

**Affiliations:** 1 Centre of Precision Rehabilitation for Spinal Pain, School of Sport, Exercise & Rehabilitation Sciences, University of Birmingham, Edgbaston, Birmingham, UK; 2 School of Physical Therapy, Western University, London, Ontario Canada

**Keywords:** Low back pain, Manual therapy, Expectation, Perception, Qualitative study, Thematic analysis

## Abstract

**Background:**

Chronic low back pain (CLBP) prevalence has steadily increased over the last two decades. Manual therapy (MT) is recommended within a multimodal management approach to improve pain and disability although evidence investigating the patients’ experience of MT is scarce.

**Objective:**

To explore expectations and perceptions of MT techniques in people with CLBP.

**Methods:**

A qualitative study embedded sequential to an experimental trial using semi-structured interviews (SSI) explored participants’ experiences of thrust, non-thrust and sham technique. Purposive sampling enabled variance in age and CLBP duration. An evidence informed topic guide was used. Data were analysed using thematic analysis (TA). Respondent validation and peer debriefing enhanced trustworthiness. The Consolidating Criteria for Reporting Qualitative Studies (COREQ) reported methodological rigour.

**Findings:**

Ten participants (50% male) with a mean age of 29.1 years (Standard Deviation (SD): 7.9, range: 19–43), a mean pain intensity of 4.5 on a Numeric Rating Scale (NRS) 0–10 (SD: 1.5, range: 2–7), a mean Oswestry Disability Score (ODI) of 9 (SD: 4.6, range: 2–17) and a mean Tampa Scale of Kinesiophobia (TSK) score of 38.6 (SD: 4.8, range: 30–45) participated. Four themes were identified: understanding of pain; forming expectations; perception of care; re-evaluation of body awareness and management. Understanding of CLBP is formed by an individuals’ pain perception and exchange with social environment. This, combined with communication with physiotherapist influenced expectations regarding the MT technique.

**Conclusion:**

Expectations for MT were formed by an individual’s social environment and previous experience. A treatment technique is perceived as positive if its characteristics are aligned with the individual’s understanding of pain and if care is delivered in an informative and reassuring manner.

**Supplementary Information:**

The online version contains supplementary material available at 10.1186/s12891-021-04251-3.

## Highlights

- Participants’ understanding of low back pain was shaped by their own perception of pain and social environment.
- Previous experience of manual therapy and understanding of pain formed expectations prior to manual therapy.
- Meeting participants’ expectations, regarding the effect of manual therapy and interaction with therapist, enhanced satisfaction with care.
- The thrust-manipulation technique elicited more equivocal reactions than the non-thrust-mobilisation technique.

## Introduction

The point prevalence of chronic low back pain (CLBP) is estimated as 19.6% between 20 and 59 years of age [1]. Research investigating factors for developing CLBP continues to be of interest [2, 3]. A relationship between sociological and psychological factors e.g. self-efficacy [4], fear avoidance [5] and catastrophising [6], has been established.

Alongside significant impairment in daily tasks and social interaction [7, 8], recent research reports a change in sensory and emotional perception in people with CLBP [9, 10]. Impaired discrimination of touch on the affected level due to a dysfunctional body image has been observed [9]. A strong identification with pain occurs as symptoms persist, leading to a change in interaction with the environment e.g. social isolation [10]. Expectations on health care practitioners to alleviate the burden of musculoskeletal complaints are high and complex [11]. Influencing factors for patient satisfaction in management of CLBP include reduction in pain, regaining perception and function [12, 13].

Manual therapy (MT) in combination with education and exercise is recommended for management of CLBP [14]. MT contributes to the restoration of tactile acuity through the stimulation of the somatosensory cortex [15]. A combination of peripheral, spinal and supraspinal effects of MT has been proposed [16]. Significant changes in pain are reported with manipulation (thrust) and mobilisation (non-thrust) techniques with an additional effect of thrust-manipulation on disability [17]. However, no consideration of patients’ underlying beliefs and knowledge has been explored during the efficacy trials [18]. A comprehensive understanding of the patient’s neurophysiological, psychological and sociological perspective may assist in selecting the most appropriate technique [19, 20].

Maiers et al. [21] investigated the perception of MT and exercise in older adults. Perceived change in symptoms was only valued by 17% of 222 participants, whilst more than half valued interaction and relationship with the therapist. These findings underpin the importance of understanding which factors contribute to high satisfaction in treatment [21]. Evidence supports the positive influence of meeting patients’ expectation to enhance treatment satisfaction [12, 22]. However, meeting patients’ expectation did not influence pain, disability and the perception of recovery in 149 LBP patients receiving MT [23]. An in-depth understanding of how expectations towards MT are formed and its effect on treatment perception is missing. To date no qualitative study has investigated expectations of MT in CLBP.

The aim of this study was to explore expectations and the perceptions of MT in people with CLBP.

## Methods

### Study design

This qualitative study was embedded sequentially following a three-arm experimental efficacy trial, with 1 week wash out between interventions, conducted between the 01/06/2019 and 26/07/2019 at the Centre of Precision Rehabilitation for Spinal Pain (CPR Spine), University of Birmingham, UK. The three visits in the efficacy trial consisted of assessment of pain [(Numerical rating scale (NRS) and pain pressure threshold (PPT)], range of motion (RoM) and muscle stiffness (shearwave elastography) before and after the application of thrust-manipulation, non-thrust-mobilisation and sham technique.

### Participant recruitment and randomisation

Participants in the efficacy trial were recruited via posters in local public areas (University Campus). Potentially eligible participants (*n* = 51) were assessed based on the following criteria.

Inclusion criteria: 18–55 years, CLBP > 12 weeks, NRS > 2/10. Exclusion criteria: received any form of treatment with exercise and hands-on component (eg. physiotherapy, chiropractic, massage) in the previous 12 weeks; LBP with leg pain, indicators of red flags e.g. night pain, a history of inflammatory rheumatic disease, infectious disease, neuromuscular disease, vascular disease, connective tissue disease, osteoporosis, severe disabling pain, morbid obesity, and pregnancy.

Thirty-six participants met the criteria and were included in the efficacy trial. Block randomisation was used to ensure equal technique allocation [24]. Participants drew the sequence of the techniques from concealed envelopes. The assessors were blinded to technique allocation.

**Fig. 1:**
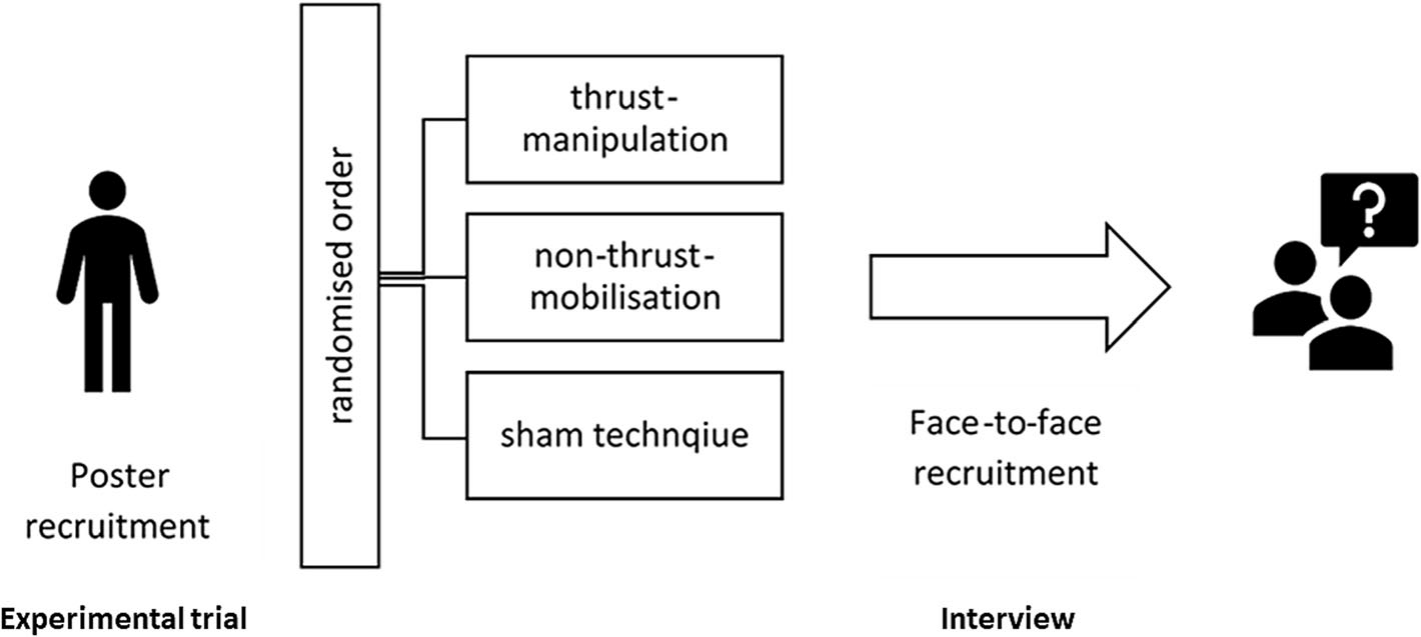
Recruitment process and trial procedure

An overview of the recruitment process and trial procedure can be viewed in Fig. 1. Further report on methodological rigour in line with Consolidated Criteria for Reporting Qualitative Research [25] is displayed in supplementary file 1.

### Intervention

The techniques were performed by an experienced manual therapist (AP). The participants were positioned in a side-lying position. Participants were lying on the asymptomatic side, If unilateral pain was present. Techniques were applied to the joint (lumbar spine levels 1–5) which on pre-thrust examination was deemed the stiffest or most hypomobile.

Thrust-manipulation: a single rotational thrust was performed [26].

Non-thrust-mobilisation: 3 sets of 20 s oscillatory rotational mobilisation was performed [27].

Sham technique: physical contact between the therapist’s hand and the participant’s lower back was established but no rotational movement was performed. Participants were maintained in a side-lying position for 30 s [28].

### Interview

Following the final visit in week 3, a 45–60 min interview was conducted by AP. From 36 participants who commenced the trial, 33 completed and were eligible for inclusion in the qualitative study. 16 participants met the language requirements (fluent in English) for participating in a semi structured interview. 10 of which were purposively sampled, thus allowing for the development of meaningful points of similarity and difference. The purposive sampling strategy aimed at investigating a sample with similar physical attributes (CLBP), but a variance in gender and duration of LBP [29, 30] This ensured exploration of various perspectives regarding the perception and expectation towards MT.

Interview participants were encouraged to take notes about their expectations and experience of the technique after each visit. The notes could be revised before taking part in the interview. The interview took place in a quiet room separate from the main trial setting.

### Reflexivity and rapport

Participants were aware of the profession and experience of the researcher (AP). Furthermore, participants were informed about the rationale of the study (mixed methods study within a MSc research project) and objective of the interview. The researcher aimed at establishing rapport through informal (work, hobbies) and formal (aim and content of the study) conversation [31]. Participants were educated about the two distinct roles of AP, as a manual therapist and interviewer in this study. In this way, the researcher developed a closer relationship with the participants and gained a unique perspective of their experience.

### Interview topic guide

The interview topic guide (Supplementary file 2) was co-designed by the investigators (all with expertise in musculoskeletal physiotherapy and some qualitative research) for credibility and informed by an in-depth analysis of qualitative literature on the lived experience of back pain. The interview explored changes in expectation and the perception during the course of the three visits [7, 10, 13]. Following two pilot trials, two further prompts were added to explore how previous experience with hands-on therapy influences the perception of the current techniques. *(Can you describe the setting and purpose of your last hands-on treatment? Did this trial change your perspective on hands-on treatment?)*

Open-ended questions encouraged the interviewees to guide the discussion without interfering with the thought process to enhance richness and depth of the data [32]. This allowed a better insight to the values and benefits of the interventions, important for its further implementation [33].

### Data collection and analysis

Interviews were audio-recorded and professionally transcribed verbatim before proceeding to thematic analysis [34]. AP analysed and coded the transcripts in an iterative process. Themes developed from a pattern of similar codes of the first transcripts. Codes of further transcripts supported or altered existing themes as well as created new ones [35]. In the course of re-reading the content several times, themes were scrutinised against the content of the transcripts until modification of the themes ceased [34]. Respondent validation minimised researcher bias and subsequently AP presented the results back to the research team for further discussion until peers agreed on the terms and relationship of themes [36]. The interpreted data was analysed for coherence and minor wording changes were undertaken to further improve credibility of the findings [37]. Organisation of data occurred in Microsoft Excel. Quotations were used to illustrate and support themes.

### Ethical approval and considerations

Written informed consent and risk assessment ensured willingness and safety of the participants. Participants had the right to withdraw from the study at any given time up to 1 month after data collection. Data was kept confidential and was stored together with the document linking name and number of participants in the principal investigator’s office, only accessible via password. Demographic data and quotations are presented in a way, which prevents identification of individuals. Ethical approval was granted by the University of Birmingham Ethics Review System (ERN_19–0167).

## Results

Ten participants participated in the interviews, 5 female and 5 male participants with a mean age of 29.1 years (Standard Deviation (SD): 7.9, range: 19–43). Participants had a mean pain intensity of 4.5 out of 10 (SD: 1.5, range: 2–7) on the Numeric Rating Scale (NRS) as well as a mean disability score of 9 (SD: 4.6, range: 2–17) in the Oswestry Disability Index and a mean score of 38.6 (SD: 4.8, range: 30–45) on the Tampa scale of Kinesiophobia Questionnaire. History of LBP ranged from 3 months to more than 10 years.

**Fig. 2:**
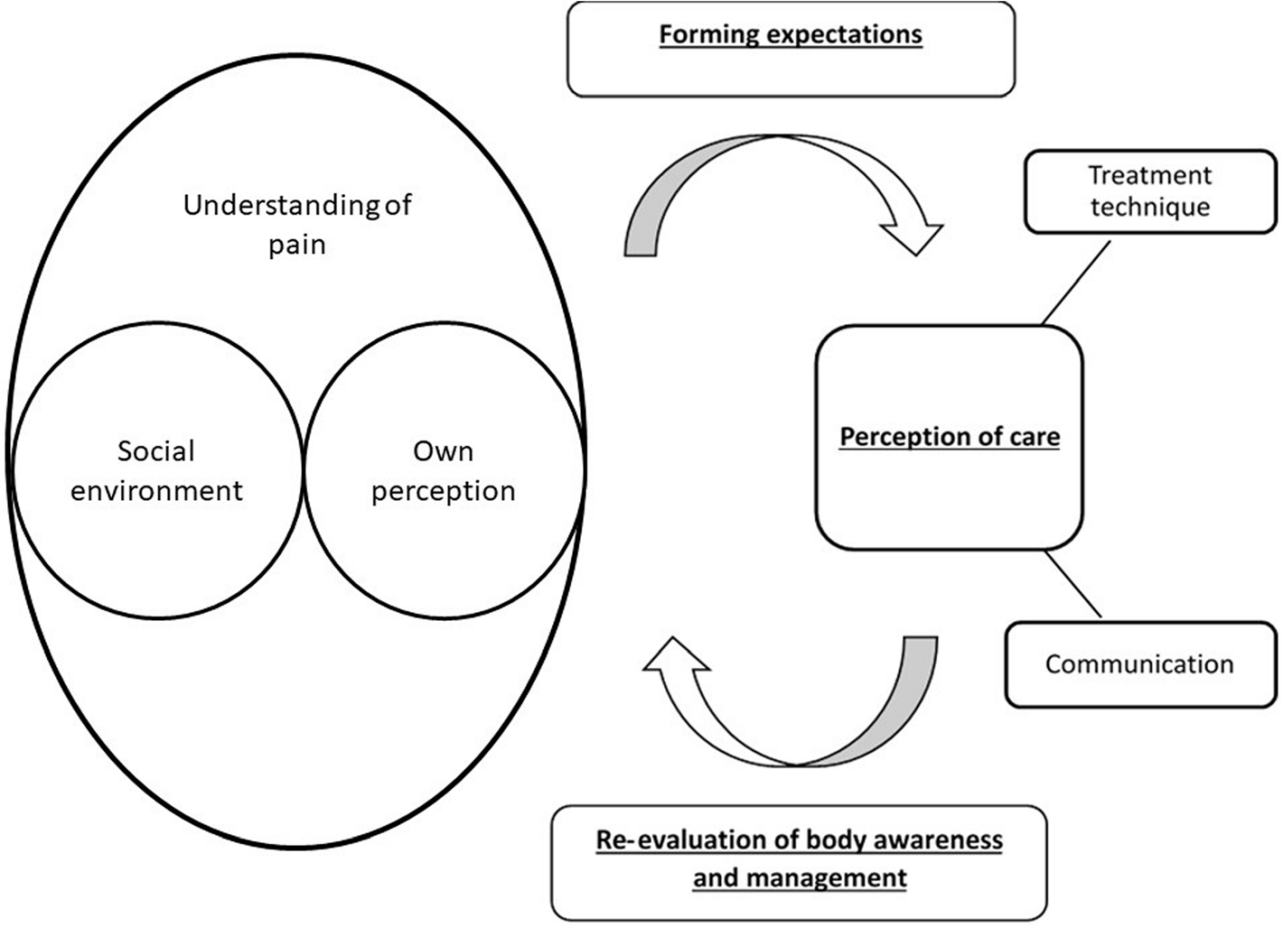
Main themes

Following four main themes were identified in the data (Fig. 2): Understanding of pain, the perception of care, forming expectation and the re-evaluation of body awareness and management. Two of the four main themes derived from subordinates. The understanding of pain was formed of the subordinates: social environment and the own perception. The perception of care derived from the subordinates: communication and type of MT technique. A detailed list of themes, codes and quotes can be found in supplementary file 3. Supplementary file 4 illustrates how themes developed over course of data analysis and peer debriefing.

The cross-case comparison table (Table 1) shows similarities and differences among participants. It also highlights the influence of experience and expectation on the preference for a treatment technique.

**Table 1:** Examples from cross-case comparison

Parti-cipants	Understanding of pain	Forming expectation	The perception of care	Re-evaluation of body awareness and management (technique preference)
01	Self-manipulation and movement help; experience with injuries and rehabilitation in the past; biomedical background to pain	Communication adapted to individuals background and knowledge, treatment short and effective on pain, Physio will help	Well- detailed explanation Professional behaviour; Well-organised and smooth procedure	Frequency of pain reduced after thrust-technique (pressure release) Additional rotational spine movement is required for effect (thrust-technique)
04	Rich experience of hands-on treatment; perceives two types of pain (central localized, vs diffuse) Agrees on psychological component to pain; life-long process of realignment	Proper explanation to techniques and origin of CLBP; Influencing the “sense of wrongness” in the back	In general, too short and unspecific to be effective; Singular treatment approach is not sufficient,	A slight shift in pain occurred during thrust-technique; however, acquired active coping-strategies are more helpful (no preference)
06	Pleasant experience with physiotherapy (mainly exercises) Asymmetry and alignment issue Stress-induced pain	No high expectation on solving underlying pain drivers	Enjoyable interaction with Physiotherapist Apprehensive to thrust technique; pleasant experience with non-thrust technique	Most change in pain immediately after treatment and during activities; similar to exercise from previous physiotherapist (non-thrust technique)
08	Self-manipulation and strength training help; Weak and “out-of-place” spine drives the pain;	Scientific approach expected; influencing “stiffness and knots” Detailed explanation of procedure	Comfortable communication and touch; perception of professional manner and sufficient explanation; nice stretch with both techniques	Explanation impacts outcome; Unspecific feeling of change through technique Something has moved back into place (thrust-technique)

The individual understanding of pain was formed of the subordinates:

### Understanding of pain

From the initial onset of their LBP, participants intended to build up an understanding of movements or activities, which contributed to a decrease or increase in their pain.

“*… I have to be careful. I always try to wear a sport belt or whatever, to keep the spine (aligned). It’s just sometimes you do forget and you make a sudden movement, it can go.”* P09.

*“...trying different positions to make my back crack because that’s what gives me relief most of the time.”* P07.

#### Own perception

Certain beliefs and concerns may have been responsible for driving the LBP and were identified in the interview. The perception and belief of a “misaligned spine” was common among participants and may be responsible for the persistence of pain. Participants also raised concerns about not getting enough rest or lacking time to exercise properly.

*“… if I were to run, I would have that, ‘oh my back is hurting’ kind of pain, a more intense pain, the perception that something is out of place.”* P08.

#### Social environment

These beliefs and perceptions have developed not only internally, but also as a result of external factors. Participants knowledge and awareness of back pain was shaped by prior experience with health care practitioners including physiotherapists and general practitioners.

*“...that’s one of the things that she (physiotherapist) told me, like gave me some exercises I can do, with movement, to loosen things up a bit. I think that is probably what made it (non-thrust mobilisation technique) pleasant, because it reduced some of the stiffness or the soreness in the muscle.”* P06.

In addition, people with LBP exchanged experiences and opinions and contributed to understanding of pain informed by the social environment. Statements like “knots in back”, “stiff back” and “entrapped nerve” sticked to and modified several participants’ cognition and awareness. It may have also influenced the way an effect of the treatment was perceived by and communicated among individuals.

*“… I think these terms that you hear people use, like I’ve got a knot in my back; you just assume it’s that.”* P08.

*“… I’ve heard that people like yes, they crack and they feel relieved after, they feel like new. I don’t know if that’s true.”* P10.

### Forming expectations

Prior to the MT sessions, participants developed expectations on what will occur during the visit. Depending on their understanding of pain and explanation of the physiotherapist, expectations were met in different ways considering dosage and characteristic of the technique.

*“… It was okay but I expected that one to work, so my expectation was not too high because I felt mobilising 20 times, that would actually cause something to be better, and the effect I got was actually good, equal to the expectation I have, so I was not too excited.”* P02.

The majority of participants associated muscles with the source of pain and movement being the most beneficial self-management strategy. Thus, techniques without movements (e.g. sham therapy) did not fulfill the expectations of many participants.

*“… I just did not feel like it did anything, it was like a bad hug! (Laughs) It just did nothing. As is my way, I just almost completely eliminated that from being of any relevance at all. I did not feel like anything had happened.”* P04.

Conversely, participants with the belief that their spine was not aligned, a thrust-manipulation accompanied by a cavitation sound achieved satisfaction. Especially if positive experiences with prior self-manipulation existed, regardless of the duration of the technique or effect. When asked why a thrust-technique has more effect on him than a non-thrust technique, a participant explained:

*“… I guess maybe because of the cracking. I think I often, personally associate, if I, like I was saying, if I crack my back, when I’m working out or something, then for me that’s like oh, okay, it’s fine, I’ve cracked it and there’s no pain at the moment, so it’s okay.* “P07.

### The perception of care

Participants tended to report positive or negative experiences of care, depending on how they perceived the MT technique and the communication between them and the physiotherapist. A preferred treatment technique was selected if a positive change in pain and function was felt (Table 1).

#### Communication

Communication was appreciated in an informal and formal way, provided the content was adapted to the individual’s knowledge and interest. Participants were comforted and reassured by engagement through informative and casual conversation.

*“… I would say like for a person like me who doesn’t know too much about these things, use more simple language rather than describe things with the right terminology because I won’t understand.”* P10.

*“… Oh, cool. He’s touching my L4, L5. So, I knew you were touching L4, L5 because you told me, whereas with other doctors, they don’t tell me, L4, L5.”* P01.

Furthermore, in some cases, specific information led to a deeper understanding and improved compliance with the procedure. Two participants described an effect on their mind set before receiving the treatment. Potentially also showing an impact on the immediate outcome after the treatment.

*“… I think the more discussion you can have with someone, the better the treatment will probably be because if you can understand more about where your pain is coming from and how the treatment is working, then I think it would be more beneficial for the person receiving the treatment.”* P07.

#### The perception of MT technique

Likes and dislike about effectiveness and comfort were reported for all three techniques. The cross-case analysis table elucidates the perception of and preference for MT. Thoughts and opinions differed according to their understanding of pain and previous experience with hands-on therapy. The thrust-manipulation evoked the most ambiguous responses from “energy release” to “a feeling something is braking”.

*“… Then with the other one, the one with the click (thrust-technique), that was the most anxious, just purely because I didn’t know what to expect. He was like, going to do this click and it was just like, you know, this could go wrong! I’ve never had anything like that before.”* P03.

*“… The difference between manipulation and that (non-thrust technique) was that in the manipulation you just went that extra bit and cracked it.”* P01.

Participants reported a less vigorous movement with the non-thrust manipulation compared to the thrust-manipulation. The non-thrust manipulation elicited a more familiar sensation, similar to Pilates stretches or pre-exercise warm up.

*“… but the second one (non-thrust mobilisation), it did kind of … It’s a moving Pilates where you’re lying on your side, you tap your heel five times up to the front, point your toe and tap it five times to the back, doing an N-shaped arch over it.”* P04.

### Re-evaluation of body awareness and management

Participants tended to immediately reassess their pain after the treatment to observe and sense any form of change to movement or pain.

*“… I think that’s why I felt the second time when you did the manipulation, I instantly felt, because I think if you see the intensity or whatever, when I did the extension I didn’t feel any pain at all straight after. Zero. Nothing.”* P01.

Several participants acknowledged the importance of change in muscle looseness in their daily activities after treatment e.g. washing dishes, going to the gym.

*“… I think since after the second one (sham therapy), I went to work the same day, I went to work in the evening and I felt that it was easier to lift certain things.”* P10.

## Discussion

This was the first qualitative study exploring expectations and the perceptions of people with CLBP with low to moderate pain, disability and fear avoidance of different MT techniques. The findings exposed a relationship between the understanding of pain and how MT was perceived. Expectations formed by an individual’s medical and social background, as well as interaction with the therapist may have influenced how the techniques were experienced. Treatment techniques tended to be favoured based on prior personal experience and beliefs of the participants as well as perceived change immediately after its application.

### Understanding of pain

Perspectives and opinions about what has caused and driven back pain varied considerably. Biomedical (P01, P08) and psychosocial (P04, P06) causal factors were mentioned. Participants in this study appeared to be very much aware and in control of aggravating and easing activities. Qualitative studies, investigating beliefs and understanding of CLBP in participants with high levels of pain and disability described a more substantial alienation between mind and body, resulting in cognitive and emotional exclusion of painful body areas [8].

The perception of weak and misaligned spine may have been due to an alteration of the somatosensory cortex through long exposure to pain [15]. Another explanation may have been an exchange of beliefs with friends and family going through a similar experience or even health care professionals [38]. Certain attitudes and terminology like “knots in my back” (P08), tended to reinforce this understanding. To put this matter in relation to MT: How individuals perceived themselves may have not only influenced the understanding of back pain but also the perception of therapy.

### Forming expectations

A determining factor for the existence and development of expectation was previous experience [39]. Extensive and unsuccessful previous experience with therapy tended to lower expectations (P04). Recommendations for specific therapy or techniques from social environment (friends and family) or previous positive experience appeared to raise expectations (P07). Participants with higher levels of disability also showed an increase in expectation prior to treatment [40].

Participant’s expectation of type and applied location of MT depended on what was believed to be responsible for the pain. However, more attention was given to factors unrelated to the technique. An informative and empathic style of communication and explanation of possible reasons for pain persistence, was expected to be obtained. The importance of expectation regarding either to communication or to the performance of the technique may vary between individuals and sessions [41].

### The perception of care

Participants reported a raise in body awareness and sense of psychological comfort through LBP-related positive and informative communication. Thus, it may have effected how treatment was ultimately perceived. Bialosky et al. [42] reported a significant correlation between (positive, negative, neutral) instructions regarding the effect of the treatment on pain and pain expectation. However, no notable change in outcome within the different instruction groups was observed. As the trial by Bialosky et al. only included healthy participants, it is difficult to say whether the absence of change in outcome is also applicable in people with CLBP with additional factors (hypervigilance, fear-avoidance, etc.) being present.

Bishop et al. [12] indicated a potential for participants naïve to MT not being able to differentiate between thrust and non-thrust techniques. Yet, the majority of the interviewed participants had no prior knowledge of MT and were able to give a rich insight to their perception during the short application of the technique. Especially the thrust technique elicited strong immediate responses in some participants. Two possible factors may have contributed to this phenomenon. Firstly, the cavitation sound elicited a sense of release and has been associated with a belief of realignment and correction [43]. Secondly, individuals perceived the “extra bit” of movement (P01), as an unfamiliar sensation and beyond their muscular control. This sense of additional range and input during the thrust created a distinct sensation, which could have also been perceived as uncomfortable or intimidating.

### Re-evaluation of body awareness and management

Participants tended to reassess their perception of pain and function immediately after the technique was applied. In this first process of re-evaluation, participants evaluated if the perceived effect had met their expectation in terms of pain relief and regain of function. Depending on pain level and past MT experience, this could have had a lower or higher influence on treatment satisfaction. Subsequently participants re-evaluated the perception of care and therapist relationship, which potentially contributed more to overall treatment satisfaction [21]. A technique was worthwhile, if the effect was comparable to previous MT experience (P06) or self-acquired coping strategies (P04).

### Strengths and limitations

The methodology used allowed a rigorous investigation of an individuals’ verbal account. Rigor was established through a transparent coding process and development of themes. Participants were given a chance to review the codes and interpretations of their verbal account via email. Half of the participants responded to the credibility checks with no further comments to the interpreted data. The response rate of credibility checks may have been increased by an additional incentive for reviewing the data or a fourth face-to-face visit containing a discussion of the interpreted verbal account. With special interest in the spine and experience in reviewing and conducting qualitative research, co-investigators enriched credibility through emphasising the importance of an iterative and honest coding process.

Field notes before and after the treatment techniques may have captured expressions and emotions in a more authentic way and would have served to enhance credibility of the study through triangulation of data [44]. Data saturation could not be considered, as the transcribing process commenced after the completion of the last interview. It is therefore unclear if sample size was large enough to provide data saturation.

The role of the researcher (AP) as therapist and interviewer may have influenced statements regarding comfort and efficacy of MT techniques as well as interaction and communication with participants. This circumstance may be viewed as a strength (richer insight through a longer bonding process) or a weakness (avoidance of critique).

### Implications for research and practice

Study findings illustrate the importance of investigating a patient’s belief and understanding of their LBP presentation. A thorough patient history including past experiences with manual therapy may add value to a clinician’s decision-making process. A disparity between expectations of the physiotherapist and patient may lead to an unsatisfactory treatment experience [39]. The perception of a treatment technique is unique to the individual and certain factors (e.g. comfort, invasiveness, performance) may influence preference. Depending on individual’s own understanding of LBP, a particular MT technique may or may not be an appropriate choice. E.g., due to the diverse responses of the thrust-technique in this study (ranging from instant pain relief to uncomfortable and intimidating technique), it is recommended to discuss beliefs and actual effects of the thrust technique prior to its application.

Further qualitative research is required to investigate expectations and the perceptions of MT in a population with higher levels of disability, pain and fear avoidance. Findings are expected to vary from this study, as participants may show higher expectations towards treatment [40] and a change in body perception and cognition [8].

## Conclusion

Perception and expectation of a MT technique may be influenced by an individuals’ understanding of CLBP as well as instruction and application of the technique by the therapist. Perception of pain and exchange with the social environment could play an important role on how back pain was understood. Thus, expectation prior and perception during the technique varied among individuals in this study. Participants responded differently to the thrust technique than the non-thrust technique, likely due to a consequence of the cavitation sound and speed elicited by the technique. The re-evaluation of pain, disability and overall experience tended to occur immediately after the technique and supported the decision of whether the MT technique was worthwhile.

## Supplementary Information

**Additional file 1.**

**Additional file 2.**

**Additional file 3.**

**Additional file 4.**

## Data Availability

The datasets used and/or analysed during the current study available from the corresponding author on reasonable request.
