# Supplementary material for: Exploring expectations and perceptions of different manual therapy techniques in chronic low back pain: a qualitative study
Source: BMC Musculoskelet Disord. 2021 May 14;22:444. doi: 10.1186/s12891-021-04251-3 (PMC8122532; doi:10.1186/s12891-021-04251-3)
Supplement: Supplementary file 1 — Additional file 1. [file 12891_2021_4251_MOESM1_ESM.docx]

| **Supplementary File 1**  Consolidate Criteria for Reporting Qualitative Research (COREQ) Checklist | | | |
| --- | --- | --- | --- |
| Number/ Item | Guide question | Description | Location in Article |
| **Domain 1: Research team and reflexivity** |  |  |  |
| *Personal characteristics* |  |  |  |
| 1 Interviewer/facilitator | Which author/s conducted the interview? | XX | Methods |
| 2 Credentials | What were the researcher’s credentials? | XXXXXXXXX  XXXXXX  XXXX  XXXX | N/A |
| 3 Occupation | What was their occupation at the time of the study? | Physiotherapist (musculoskeletal) | Methods |
| 4 Gender | Was the researcher male or female? | Male | Methods |
| 5 Experience and Training | What experience or training did the researcher have? | XX had no previous experience with interview and undertook 2 pilots before commencing with participants | Methods |
| *Relationship with participants* |  |  |  |
| 6 Relationship established | Was a relationship established prior to study commencement? | XX – performed 3 MT techniques with the participants prior to the interview | Methods |
| 7 Participant knowledge of the interviewer | What did the participants know about the researcher? | Participants were informed about prior experience with the MT techniques. Participants were briefed on the nature of the study (embedded qualitative trial for the MSc theses) They understood that the researcher’s aim was to evaluate their perceptions and thoughts on the different techniques | Methods |
| 8 Interviewer | What characteristics were reported about the interviewer? | AP has a special interest for CLBP and takes the position of an interpretivist researcher | Methods |
| **Domain 2: study design** |  |  |  |
| *Theoretical framework* |  |  |  |
| 9 Methodological orientation and theory | What methodological orientation was stated to underpin the study? | Interpretative phenomenological analysis | Introduction  Methods |
| *Participant selection* |  |  |  |
| 10 Sampling | How were participants selected? | Purposive sampling (gender matched and maximum variance in low back pain duration)  From a homogenous CLBP sample without comorbidities or undergoing treatment | Methods |
| 11 Method of approach | How were participants approached? | Quantitative trial: poster advertisement on the Campus of the XXXXXXXXXXXXX. Followed by recruitment via email  Qualitative trial: face-to-face recruitment at the beginning of the 1. MT session. | Methods |
| 12 Sample size | How many participants were in the study | 10 | Results |
| 13 Non-participation | How many people refused to participate or dropped out? Reasons? | After agreeing to participate and signing a written consent-form, none of the 10 participants refused to participate or dropped out. | Results |
| *Setting* |  |  |  |
| 14 Setting of data collection | Where was the data collected? | The interviews were conducted within the facilities of the CPR Spine department in the XXXXXXXXXX in a quiet room. | Methods |
| 15 Presence of non-participants | Was anyone else present beside the participants and researchers? | No | Methods |
| 16 Description of sample | What are the important characteristics of the sample? | The sample consist of 5 male and 5 female participants with CLBP duration from 3 months to over 10 years and age ranging from 19 to 43 with a mean age of 29 | Results |
| *Data collection* |  |  |  |
| 17 Interview guide | Were the questions, prompts guides provided by the authors? Was it pilot tested? | The semi-structured interview guide (Supplementary File 2) was developed after a thorough investigation of current literature on expectations of hands-on techniques as well as lived experience with CLBP. Further adaptions to prompts were made after 2 pilot interviews | Methods  Supplementary File 2 |
| 18 Repeat interviews | Were repeated interviews carried out? If yes, how many? | No | n/a |
| 19 Audio/visual recording | Did the research use audio or visual recording to collect the data? | The interviews were audio-recorded. | Methods |
| 20 Field notes | Were field notes made during and/or after the interview? | No | n/a |
| 21 Duration | What was the duration of the interviews? | 30-40 minutes | Methods |
| 22 Data saturation | Was data saturation discussed? | Interviews were sent to transcription services after the last conduction. Thus, data saturation could not be considered. | Methods |
| 23 Transcript returned | Were transcripts returned to participants for comment and/or correction | No | n/a |
| **Domain 3: analysis and findings** |  |  |  |
| *Data analysis* |  |  |  |
| 24 Number of data coders | How many data coders coded the data? | Coding of data was performed by one researcher XX | Methods |
| 25 Description of the coding tree | Did authors provide a description of the coding tree? | Supplementary file 4 displays the different stages of theme development | Supplementary file 4 |
| 26 Derivation of themes | Were themes identified in advance or derived from data? | XXXXXXXXXXX discussed the derivation of themes separately and undertook modifications to the relationship of themes. A detailed description of coding process can be viewed in supplementary file 3 | Methods  Supplementary file 3 |
| 27 Software | What software, if applicable, was used to manage the data? | Data was coded and themed in Microsoft Excel | Methods |
| 28 Participant checking | Did participants provide feedback on the findings? | Yes, before commencing with the constant-comparison analysis, the respondents validated the transcribed and interpreted data. | Methods |
| *Reporting* |  |  |  |
| 29 Quotations presented | Were participant quotations presented to illustrate the themes/findings? Was each quotation identified? | Yes, superordinate and subordinate themes and their relationship were supported by 1-2 quotations from participants. The quotation was identified by the confidential participant number. | Results |
| 30 Data and findings consistent | Was there consistency between the data presented and the findings? | Data and findings were discussed within a focus group of two uninvolved and one involved researcher XX Independent check for consistency and coherency was performed independently by another researcher (NH) with qualitative research experience before publication | Methods |
| 31 Clarity of major themes | Were major themes clearly presented in the findings? | Yes, Major themes were described and supported by participant’s quotations. | Results  Figure 2 |
| 32 Clarity of minor themes | Is there a description of diverse cases or discussion of minor themes | Yes, current literature on expectations and perceptions of hands-on therapy was discussed and compared to the findings. A cross-case-comparison table was developed (Table 3) | Findings  Table 3 |
