# Supplementary material for: Exploring expectations and perceptions of different manual therapy techniques in chronic low back pain: a qualitative study
Source: BMC Musculoskelet Disord. 2021 May 14;22:444. doi: 10.1186/s12891-021-04251-3 (PMC8122532; doi:10.1186/s12891-021-04251-3)
Supplement: Supplementary file 2 — Additional file 2. [file 12891_2021_4251_MOESM2_ESM.docx]

**Supplementary File 2**

Interview topic guide

1. Expectations regarding manual therapy and study design:
   1. Have you had any previous experience with manual therapy prior to the participation in this study?
      1. If yes, can you describe the setting and purpose?
   2. What were your expectations and thoughts before the first visit?

(prompts: anxious, hopeful, impartial)

1. Perception during the MT session:
   1. Please tell me about your experience during the three MT sessions.
      1. Which technique did you like best? Why did you prefer it?
      2. Which technique did you like least? Why were you not fond of it?
      3. Can you describe your perception during the three trials?
      4. What did you think was happening to your body?
   2. How did you find the setting of the study?

(prompts: length of MT and assessment; communication and explanation)

- 1. Please give some insight on the relation between the physiotherapist and yourself (prompts: important for effect of care? what was good/bad about it? recommendations for further visits)
  2. Did this trial change your perspective on MT?

1. Personal beliefs about symptoms:
   1. If you feel comfortable enough, please share your feelings and beliefs about your back-pain symptoms.
      1. What has helped you cope with the back pain?
      2. What aggravates it?
   2. Has this study changed your knowledge and view of your symptoms?
      1. If so, what do you take away from it?
   3. What will be your plan for the near future?
