# Supplementary material for: Exploring expectations and perceptions of different manual therapy techniques in chronic low back pain: a qualitative study
Source: BMC Musculoskelet Disord. 2021 May 14;22:444. doi: 10.1186/s12891-021-04251-3 (PMC8122532; doi:10.1186/s12891-021-04251-3)
Supplement: Supplementary file 3 — Additional file 3. [file 12891_2021_4251_MOESM3_ESM.docx]

| Superordinate Theme 1 | Subordinate Theme | Code | Example from transcript | **Related superordinate Theme 2**  **(Forming Expectation)** |
| --- | --- | --- | --- | --- |
| **Understanding of pain "** | Perception of self | What helps it | “…so I don’t really do it to the extent where you do completely rotate it, it doesn’t happen, and I think that movement is required. I actually need another partner or something to come and do that in the gym, which I don’t get to, so I think if you do that as a stretch or a rehab, it would help in the long run to mobilise my spine.  **P5 (190-194)** | Treatment technique  (Manipulation) |
|  |  |  | “It was okay but I expected that one to work, so my expectation was not too high because I felt mobilising 20 times, that would actually cause something to be better, and the effect I got was actually good, equal to the expectation I have, so I was not too excited.”  **P7 (106-108)** | Treatment technique  (movement helps) |
|  |  |  | “Sometimes I just know I need to snap my back, not because my back is in pain but because it feels wrong. I don’t know quite what’s going on with my body interpreting things then…”  **P 18 (162-164)** | Treatment technique (Manipulation) |
|  |  |  | Regarding the effect of explanation and relationship to comfort:  “No, because I knew what I signed up for. It’s that the first time was more unpleasant, but I mean, that’s not you doing that intentionally, so I don’t see that as anything that would harm that.”  **P 21 (155-157)** | Communication  (Formal) |
|  |  |  | “I’ve found with all three of the therapists I’ve also been able to have an off-topic, normal conversation which I think is really nice. It’s not necessary, I don’t need to have it, but I think it is nice.”  **P20 (216-218)** | Communication  (Informal) |
|  |  | What hurts it | “I have to be careful. I always try to wear a sport belt or whatever, to keep the spine. It’s just sometimes you do forget and you make a sudden movement, it can go. That’s the physio side of it. Over the last probably, I don’t know, about five/six years, I’ve probably visited the physio about four or five times. If it gets a lot worse, I tend to, but as I said, hanging upside down technique has helped me a lot, personally.”  **P 48 (37-41)** | treatment technique |
|  |  |  | “The fact that I sit down for prolonged periods of time, which doesn’t allow me to move around as much and doesn’t allow the muscle to keep warm and active.”  **P 25 (296-298)** | n/a |
|  |  |  | “I think of all my time working in social housing, I’ve met plenty of people with micro whatever label and it just seems to be an excuse to do absolutely bugger all. Well, I think there’s a lot of joint issues with whatever it is I’ve got, keep a  that label people seem to think, I’ll just sit around and not do anything.”  **P 18 (245-249)** | Communication  (formal) |
|  |  |  | “I think because it was a sudden movement and from experience, sudden movements tend to not have a pleasant effect.”  **P 21 (112-113)** | treatment technique |
|  |  | What drives it | “It was just a birth thing, misaligned hips and that just feels like it’s right. I think there’s just slightly rubbish joints that might run in the family…”  **P 18 (233-234)** | n/a |
|  |  |  | “In my own case, I know stress has a big effect on it. I’ve gone to uni, so there’s stress all the time, everywhere. (Laughs) I think but I can’t say for sure, lack of exercise probably also has an effect on it because I’ve not been able to do proper exercise for a long time now because it just hurts way too much. I don’t know.”  **P 21 (301-305)** | n/a |
|  |  |  | “I have a feeling that it’s linked to my posture because before I received physio my posture was horrendous, my back was really arched, and I think it put far too much pressure on my lower back and I think it really didn’t help when I was dancing with bad posture.”  **P 20 (326-331)** | From social environment |
|  |  |  | I think it’s a bit of, this probably might be really wrong, I’m not sure, but I feel like, I feel that since I’ve had the twins, something is not quite right with my back. I don’t know whether my hips are out of place or something like that, but I just find that maybe is an issue.”  **P 25 (294-302)** | Relief through realignment |
|  | Social environment | Comparison with other low back pain (LBP) patients | “With similar symptoms that I’ve got, yeah. I think that’s important because everyone has got lower back pain but then it’s all, everyone has got unique types of pain or different disc issues. You want to get into the same category as other people with the same disc problem and then focus, really hone in on these guys, whatever, L5/S1, whatever ones you’re focusing on, get better results with this particular treatment. Knowing that I’m in the same category, that helps.”  **P 15 (217-221)** | Communication  (reassurance) |
|  |  | Activities impacting LBP | “For example, today I’ve hardly been mobilised at all, and last week I was sitting in a lecture theatre for five hours, so the same thing really, I’ve just been sitting, not really mobilised; whereas I’m pretty sure, I can’t quite remember but I think perhaps the first week I had been walking around before so my body was a little bit more mobilised, so it was kind of less of a shock to my muscles, if that makes sense.”  **P20 (143-147)** | Treatment technique  (change) |
|  |  | Comparison with other LBP patients | “Also, because I heard before. But, obviously, this is with chiropractors and other people that do different things, I’ve heard that people like yes, they crack and they feel relieved after, they feel like new. I don’t know if that’s true.”  **P49 (243-245)** | Relief through realignment |
|  |  | Health care practitioner (Chiropracter^a^) | “I guess probably the third one really. I know that… is it a chiropractor that does that thing with people’s back that makes it crack and stuff?”  **P 25 (251-252)** | Treatment technique  (Manipulation) |
|  |  | Familiy | “Because I’ve heard many people talking about sciatica^b^ and I think my mum had problems with that. And when she describes her pain, I thought, “Oh, that’s similar to me.”  **P49 (402-403)** | n/a |
|  |  | Health care practitioner (GP^c^) | “What happened is, the pain is normally, if you go to the GP they’ll say, “You need to rest, it takes time, it goes through the system, it will come out.” I personally believe that I probably haven’t given it enough time to heal, if there is something there. Maybe I’m doing stuff that my body doesn’t really, doesn’t want me to do basically.”  **P48 (266-270)** | Treatment technique |

| Superordinate Theme 2 | Subordinate Theme | Code | Example from Transcript | **Related super-ordinate theme 4 (Re-evaluation of body perception and management)** |
| --- | --- | --- | --- | --- |
| Perception of management | Treatment technique | Manipulation  (positive^d^) | “I think trial three (Manipulation) actually. I could feel a change from, the aggravated back pain feeling more what I would describe as crunchy and being towards the centre of my back to then being a little bit more diffuse and more in my muscles.”  **P18 (39-42)** | Change in pain |
|  |  |  | “Spine. I don’t know what a crack exactly means. I’m pretty sure it’s the same cracks like you get… is it a relief of pressure or…?”  **P5 (97-98)** | Change in pain |
|  |  |  | “…whereas the thrust one was a bigger, sharper movement. That’s what I perceived it as.”  **P20 (87-88)** | More movement |
|  |  | Manipulation  (negative^e^) | “…whereas manipulation is probably, you can do it on different sides rather than as opposed to time.”  **P5 (212)** | Short duration |
|  |  |  | “I didn’t like the third (Manipulation) one because of the thrusting, like you’re going to hear a cracking sound, it’s kind of scary and the thrusting is kind of painful.”  **P7 (109-110)** | Change in pain |
|  |  |  | “Then with the other one, the one with the click, that was the most anxious, just purely because I didn’t know what to expect. He was like, going to do this click and it was just like, you know, this could go wrong! I’ve never had anything like that before.”  **P15 (90-93)** | Apprehension |
|  |  |  | “Treatment number two (Manipulation), it did leave me in quite a bit of pain, to be honest with you, afterwards, the next day or two I was feeling, there was a lot of pain.”  **P 48 (73-75)** | Change in pain |
|  |  | Mobilisation  (positive) | Preference regarding comfort: “ I think probably the movement (Mobilisation) one, the first one, I think. I still feel conscious about the click one (Manipulation), but I think the movement one, just to get a bit of flexibility around that lumbar spine area, I think, yeah.  **P15 (137-139)** | Comfort |
|  |  |  | “Positive. I felt as though I could feel work being done to my back. It (Mobilisation) was done thoroughly and properly because there was a slight, I don’t know if pain is the right word, but I could feel myself being pulled and I could feel my back being worked, and then immediately after I could feel more mobilised.”  **P20 (76-79)** | Higher Intensity |
|  |  |  | “I think the one today; I would say is the one that has made my back feel like there’s pretty much no pain. Like when I did the leaning test afterwards, to touch, it didn’t hurt on my right side, there was just like a little pain on my left, just like the pain you get if you’re stretching something.”  **P22 (48-51)** | Change in activities |
|  |  |  | “Yeah, because that’s (Mobilisation) generally sort of the movement, like it fits in what I try to do to loosen up my back, before I try to do these things as well.”  **P21 (241-242)** | Change in activities |
|  |  | Mobilisation  (negative) | “There’s nothing I can say about the first treatment to be honest with you, it was just a mobilisation wasn’t it, the treatment? No, not really to be honest, there’s nothing I can give insight on that.”  **P48-(255-257)** | Not memorable |
|  |  |  | “I described it as quite pulling and bouncy. It’s had that sense of like stretching a muscle, but doing it in a wrong way.”  **P18 (49-50)** | Wrong sense of pulling |
|  |  |  | “None of the treatments made me feel worse or anything the day after. Yeah, I thought the movement one (Mobilisation) or the other one, the second one sorry, would have made me loosened it, but it kind of didn’t, it just felt tighter for some reason.”  **P15 (106-109)** | Treatment soreness |
|  | Communication | informal | “I guess the interaction with you and others was really good. Everyone was really friendly. I think that’s probably important if you’re having multiple therapy sessions, then you’d want to be able to talk to the person who is doing the therapy. I think that’s quite important.”  **P 22 (124-126)** | Interaction leads to positive effect |
|  |  |  | “I’ve found with all three of the therapists I’ve also been able to have an off-topic, normal conversation which I think is really nice. It’s not necessary, I don’t need to have it, but I think it is nice. “  **P 20 (216-218)** | Sympathy |
|  |  |  | “… being able to listen and being able to contribute, or tell if I’m wrong in terms of what I’m thinking. Mainly talk”  **P 5 (259-260)** | Sympathy |
|  |  | Formal | “I would say like for a person like me who doesn’t know too much about these things, use more simple language rather than describe things with the right terminology because I won’t understand.”  **P 49 (103-105)** | Explanation adapted to individual |
|  |  |  | “I think knowledge of what they’re doing, the fact that you explained what you were doing at every stage of what you were doing, that assisted in my confidence in knowing that you understood what you were doing and I wasn’t letting anybody just do anything. I guess the fact that you were using terminology that I’d never heard before, that made me think that you knew what you were doing.”  **P25 (207-211)** | Providing reassurance |
|  |  |  | “I think there’s something to be said as well before doing adjustments...”  **P18 (214-215)** | Preparing mindset and reassurance |
|  |  |  | “In fact, in the previous massages, obviously they probably know the basics and whatever. Whereas in this one you said, “We’re going for L4, L5^f^”, and I was like, “Oh, cool. He’s touching my L4, L5.” So, I knew you were touching L4, L5 because you told me, whereas with other doctors, they don’t tell me, L4, L5.”  **P5 (363-366)** | Gaining knowledge |
| ^a^ Chiropractor: A health profession concerned with diagnosis, treatment and prevention of mechanical disorders of the musculoskeletal system… There is an emphasis on manual treatment including spinal adjustments and other joint and soft-tissue manipulation (World Federation of Chiropractic, 2001) [1]  ^b^ Sciatica: A clinical diagnosis describing radiating pain in one leg with or without neurological deficits on examination. (Jensen et al. 2019) [2]  ^c^ GP (general practitioner): A specialist trained to work in the front line of a healthcare system and to take the initial steps to provide care for any health problem(s) that patients may have. (Olesen et al. 2000) [3]  ^d^ Positive: positive association, experience, perception or belief about a certain treatment technique.  ^e^ Negative: negative association, experience, perception or belief about a certain treatment technique.  ^f^ L4/5: the fourth and fifth spinal vertebra in the lumbar spinal column. | | | | |

References

1. Dictionary Definition WFC. Definition of chiropractic: World Federation of Chiropractic; 2001. https://www.wfc.org/website/index.php?option=com_content&view=article&id=90

2. Jensen RK, Kongsted A, Kjaer P, Koes B. Diagnosis and treatment of sciatica. BMJ 2019;19(367):I6273. https://doi.org/10.1136/bmj.I6273.

3. Olesen F, Dickinson J, Hjortdahl P. General practice – time for a new definition. Br Med J 2000;320(7231):354–7. https://doi.org/10.1136/bmj.320.7231.354
