# Supplementary material for: Exploring expectations and perceptions of different manual therapy techniques in chronic low back pain: a qualitative study
Source: BMC Musculoskelet Disord. 2021 May 14;22:444. doi: 10.1186/s12891-021-04251-3 (PMC8122532; doi:10.1186/s12891-021-04251-3)
Supplement: Supplementary file 4 — Additional file 4. [file 12891_2021_4251_MOESM4_ESM.docx]

| **Supplementary file 4**  Development and modification of the 4 major themes | | | | |
| --- | --- | --- | --- | --- |
| **Phase** | **Super-ordinate Theme 1** | **Super-ordinate Theme 2** | **Super-ordinate Theme 3** | **Super-ordinate Theme 4** |
| 1  After analysis of 5/10 Transcripts | Pain Beliefs and previous experience |  | Release effect  Movement effect  Sympathy effect |  |
| 2  After analysis of all the transcripts | External (Social environment) and internal (previous experience, beliefs) factors for understanding CLBP | Expecting certain ways of interacting and treating | Perception of change through communication and treatment | New input to knowledge |
| 3  After 2^nd^ round of transcript analysis | External (Social environment) and internal (previous experience, beliefs) factors for understanding CLBP | Expectation formed by social environment and previous experience | Perception of change through communication and treatment | Satisfaction accomplished through communication and treatment |
| 4 (final phase)  After peer-debriefing | Understanding of pain | Forming Expectations | Perception of care | Re-evaluation of body awareness and management |
